# Effect of fish oil supplement administration method on tolerability and adherence: a randomized pilot clinical trial

**DOI:** 10.1186/s40814-018-0387-0

**Published:** 2019-01-08

**Authors:** Scott S. Malinowski, Katie E. Barber, Omayma A. Kishk, Andrew A. Mays, Sara R. Jones, Adrian L. Turner, Daniel M. Riche

**Affiliations:** 1 Department of Pharmacy Practice, University of Mississippi, 2500 North State Street, Jackson, 39216-4500 MS USA; 2 University of Mississippi Medical Center, Jackson, MS USA; 3 University of Maryland Medical Center, Baltimore, MD USA; 4 Cook Children’s Medical Center, Fort Worth, TX USA

**Keywords:** Adherence, Adverse effects, Docosahexaenoic acid, Eicosapentaenoic acid, Fish oil, N-3 polyunsaturated fatty acids, Safety, Tolerability

## Abstract

**Objectives:**

Anecdotally, several strategies have been suggested in order to improve tolerability of fish oil supplements, but there is little evidence supporting any of these strategies. The aim of this study was to determine if there is a difference among four methods of oral administration of fish oil supplementation in terms of tolerability and adherence.

**Methods:**

A randomized, prospective, open-label, four-arm pilot study was conducted on 60 healthy adult subjects randomized to different fish oil supplement administration methods with (1) milk, (2) food, (3) an empty stomach, and (4) frozen capsules prior to ingestion. Each subject was instructed to take two capsules three times daily for 30 consecutive days. Adherence was assessed by pill counts. Adverse effects were assessed by survey and patient exit interview.

**Results:**

No apparent differences were demonstrated among the four administration groups in terms of adherence, reasons for non-adherence, or self-reported adverse effects.

**Conclusions:**

Method of administration did not affect rates of adherence or incidence of adverse effects in a small cohort of healthy adults taking fish oil supplement capsules for 30 days.

**Trial registration:**

ClinicalTrials.gov NCT01471366. Registered November 16, 2011.

## Background

Fish oil supplements (FOS) are an increasingly popular source of the n-3 long chain polyunsaturated fatty acids (n3PUFA) eicosapentaenoic acid (EPA, 20:5 n-3) and docosahexaenoic acid (DHA, 22:6 n-3). FOS are the most popular non-vitamin, non-mineral dietary supplement in the USA with estimated use by 7.8% of adults (an increase from 4.8% in 2007) [1].

The benefits of marine-derived n3PUFA on cardiovascular health were first proposed after the 1980 publication of the results from the Greenland Inuit Eskimo study [2]. Since that time, there have been numerous reports of cardiovascular benefit, leading to public health recommendations for increasing dietary intake of seafood rich in EPA and DHA [3, 4]. FOS benefits are also demonstrated in other areas, such as chronic inflammatory diseases, neurodegenerative disorders, and nonalcoholic fatty liver disease [5–7].

Alpha-linolenic acid is an essential fatty acid that serves as the precursor to EPA and DHA. However, the enzymatic conversion of alpha-linolenic acid to EPA and DHA is inefficient, and individuals must consume adequate EPA and DHA from dietary intake [8]. Due to inherent limitations of consuming whole fish (such as cost, access, palatability, and concerns of methylmercury contamination), FOS offer a potentially more convenient means of obtaining recommended amounts of n3PUFA. However, it is unclear if FOS provide the same benefit as whole fish consumption. Additionally, prescription FOS can be cost-prohibitive while non-prescription FOS are readily available at lower costs in general.

At recommended doses of 3 to 4 g of EPA and DHA, FOS were not associated with any serious adverse effects or detriments in clinical studies [3]. The most common adverse reactions are typically minor gastrointestinal complaints (e.g., belching, indigestion, and diarrhea) [9].

In order to consume the recommended 3 to 4 g of EPA and DHA via non-prescription FOS, a daily quantity of 6 to 10 capsules must be ingested, depending on the potency of the product being used. This high pill burden, along with the associated gastrointestinal adverse effects, can lead to nonadherence and discontinuation of the FOS therapy. Over the years, various strategies have been employed in an attempt to minimize the gastrointestinal adverse effects of FOS. Instructions to take the FOS with food or milk, and even freezing the capsules prior to administration, have become popular to improve tolerability [9–11]. There is a scarcity of published scientific literature supporting these recommendations, and any assessment of their value has been largely anecdotal. Thus, based on limited available published literature, a randomized pilot trial is needed to advance our understanding further.

The aim of this study was to determine the impact of four different administration methods of FOS capsules on tolerance and adherence.

## Methods

The study was designed as an open-label, randomized interventional trial assessing safety via parallel assignment of four different FOS administration methods. The primary outcome measures were the incidence and frequency of self-reported adverse effects associated with the FOS. Both were assessed by a questionnaire at the conclusion of a 30-day study period. The secondary outcome measure was subject adherence rate. This was assessed by final capsule count at the conclusion of the 30-day study period.

Participants were recruited from November 2011 to July 2014 by posting flyers at Walgreens and at the University of Mississippi Medical Center. These flyers were developed and distributed by the primary investigator. Those who responded to our flyers were then screened for the following inclusion and exclusion criteria in an attempt to enroll only healthy volunteers:

- Participants were included if they were between the ages of 18 and 65 years on (1) no medication or (2) only taking standard medications for the anticipated patient population (specifically oral contraceptives) or (3) had a self-reported medication-controlled chronic health condition.
- Participants were excluded if they were pregnant; nursing; incarcerated; taking any of the following medications: biologics, chronic corticosteroids, and antineoplastics, on three or more medications, or medications for self-reported uncontrolled chronic medical conditions; or reported having one or more of the following conditions: any autoimmune disorder, any uncontrolled chronic disease (e.g., hypertension, hypothyroidism, hyperthyroidism, diabetes), significant renal impairment, significant hepatic impairment, significant mental illness, or significant gastrointestinal disease.

After being screened for eligibility, subjects were contacted via phone to provide written consent and complete an initial survey in person at the University of Mississippi School of Pharmacy. The survey included a collection of basic demographic information, self-reported health conditions, and current medications. Participants were then provided a quantity of 180 non-prescription FOS capsules (Walgreens Omega-3 Fish Oil Concentrate; See Table 1). Each capsule contained 1000 mg of marine-derived fish oil consisting of 300 mg of EPA and DHA, combined. All subjects were instructed to ingest two capsules by mouth three times daily, for a total n3PUFA dose of 1800 mg daily. Each participant was provided instruction (written and oral) on how to take the fish oil capsules as specified by their respective study arm.

**Table 1:** Fish oil product details

Supplement Facts	Quantity
Calories	11
Calories from fat	9
Total fat	1 g
Cholesterol	4 mg
Natural fish oil concentrate^a^ [omega-3 fatty acids (EPA and DHA)]	1000 mg [300 mg]
Ingredients	Fish oil concentrate, gelatin, purified water USP, glycerin, natural flavors

^a^Contains anchovy, sardine, herring, and soy

Sixty participants were randomly assigned to one of the four administration method groups using a 2 × 2 block generator in an open-label allocation. Randomization was carried out by the primary investigator. The investigator responsible for randomization was not responsible for the majority of patient enrollments. The four administration method groups were as follows:

- Group 1 (“No Food”): fish oil capsules to be taken without food, on an empty stomach, at least 1 h before and 2 h after a meal (including 8 oz of water, but no dairy products within 1 h of administration)
- Group 2 (“With Food”): fish oil capsules to be taken with food (including 8 oz of water, but no dairy products within 1 h of administration)
- Group 3 (“With Milk”): fish oil capsules to be taken with milk, but no other food or beverage within 1 h of administration
- Group 4 (“Frozen”): fish oil capsules stored in a freezer until time of administration and to be taken with 8 oz of water but otherwise no food or dairy products within 1 h of administration

With the exception of group 4, all participants were instructed to store their fish oil capsules at room temperature. At the conclusion of the 30-day study period, each participant returned for an exit interview. Participants were instructed to bring their FOS study bottles and any remaining fish oil capsules to this interview. The study investigators performed a final pill count and administered an exit survey assessing outcome measures. Adherence was calculated by actual/expected pill count. As an incentive, and based on random selection, a $50 gift card was provided to one of the participants after study completion.

### Sample size

The sample size was estimated based on recruitment considerations and previous sample size recommendations. According to Julious, a minimum sample size of 12 patients is recommended per group or treatment arm based on rationale about feasibility and precision about the mean and variance [12]. Given that this study includes four treatment arms, the minimum sample size needed to be 48, so we decided to implement a higher sample size of 60 to further strengthen our study.

### Statistical analyses

Descriptive analyses were used with continuous data reported as medians or means and categorical data as percentages. Categorical data were compared using a chi-squared test. Continuous data were evaluated by ANOVA for comparisons including all four groups and by *t* test for comparison of two group means. All other data were analyzed using descriptive statistics. An alpha level of significance set at 0.05 and a *p* value < 0.05 was considered statistically significant.

## Results

**Fig. 1:**
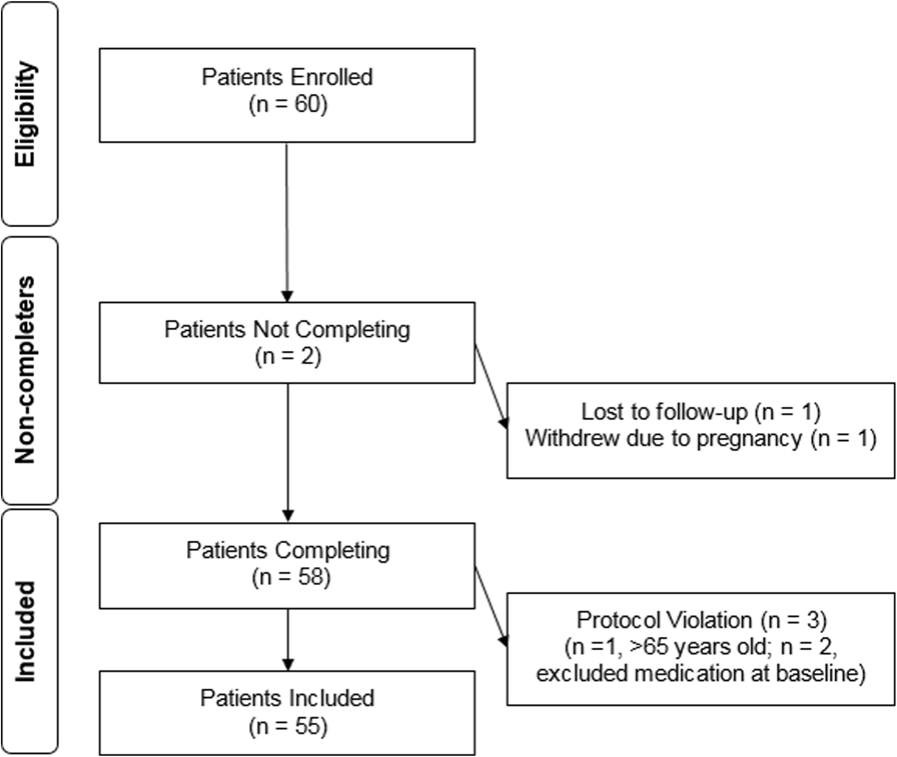
Enrollment strategy

A total of 60 subjects were consented and enrolled. Two subjects dropped out, and another three were excluded from the final analysis due to protocol violations (see Fig. 1). The remaining 55 subjects completed the study (including the exit interview), and their data was included for analysis. The baseline demographics of the participants are described in Table 2. Two subjects in the “Frozen” group did not follow instructions to freeze the capsules and therefore are included in the “on an empty stomach group.” There were no significant differences in baseline demographics between the four groups in terms of age, gender, or race. Subjects were either on no medications or standard medications [included oral contraceptives (*n* = 9) and medication for controlled conditions: attention-deficit disorder (*n* = 3), hypertension (*n* = 2), cholesterol (*n* = 2), depression (*n* = 1), migraines (*n* = 1), and testosterone (*n* = 1)]. Ten included patients reported a controlled chronic condition. The average length of enrollment in each group was 30 ± 2 days.

**Table 2:** Subject baseline demographics (*n* = 60)

Characteristic	With Food	Frozen	With Milk	No Food	*p* value
*N* (ITT)	15	13	15	17	–
Age (years) ^a^	36 ± 16	31 ± 12	29 ± 7	27 ± 4	0.06^b^
Sex (%) (M) ^c^	53	38	53	29	0.44^d^
Race (%) (CA/AA/Asian)	80/7/13	77/23/0	87/13/0	82/6/12	N/A

*ITT* intention-to-treat, *M* male, CA Caucasian, AA African American

^a^Data reported as means ± standard deviation

^b^ANOVA for between group comparisons. The only significant pairwise comparison was “Food” vs. “No Food”

^c^Ascertained by self-report

^d^Chi-squared test

**Fig. 2:**
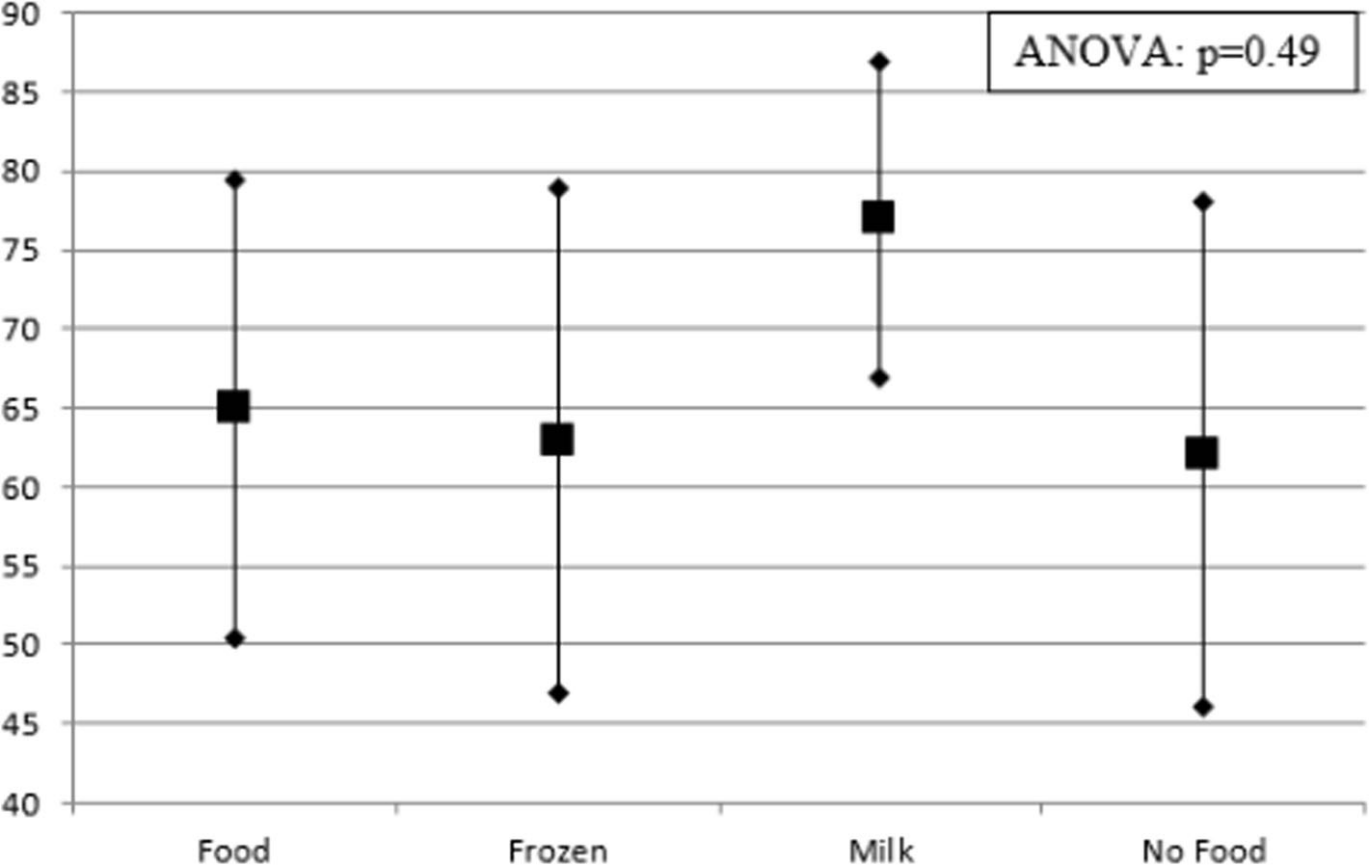
Adherence rates. Means ± standard deviation

Adherence rates, as assessed by pill counts, are described in Table 3. Complete adherence (defined as taking all 180 capsules in the study period) was low across all four groups, ranging from 8.3 to 20%. Mean adherence rates ranged from 62 to 78% across all four groups (see Fig. 2). There was no statistically significant difference in adherence rates among the groups.

**Table 3:** Adherence results by pill count

	Food (*n* = 12)	Frozen (*n* = 11)	Milk (*n* = 15)	No food (*n* = 17)
100% adherence	1	2	3	2
Overall mean adherence rate	65%	63%	77%	62%
Overall median adherence rate	75%	63%	78%	68%
All reasons for non-adherence^a,b^	Food (*n* = 11)	Frozen (*n* = 9)	Milk (*n* = 12)	No Food (*n* = 15)
Forgot	9 [81.80%]	7 [77.8%]	9 [75.0%]	13 [86.7%]
Adverse effect	1	2	–	2
Did not want to take	–	1	1	3 [20.0%]
Hard to swallow	1	–	2	1
Pill burden	5 [45.50%]	3 [33.3%]	5 [41.7%]	5 [33.3%]
Other (reason given below)				
Convenience	4 [36.4%]	2	2	4 [26.7%]
Irregular diet	1	–	–	
Milk availability	–	–	4 [33.3%]	
Freezing ability	–	2	–	
Most common self-reported reason for non-adherence^b^	Food (*n* = 11)	Frozen (*n* = 9)	Milk (*n* = 12)	No Food (*n* = 15)
Forgot	8 [72.7%]	5 [55.6%]	5 [41.7%]	9 [60.0%]
Adverse effect	1	1	–	2
Did not want to take	–	–	–	1
Hard to swallow	–	–	1	–
Pill burden	–	–	1	1
Other (reason given below)				
Convenience	2	1	2	2
Milk availability	–	–	3 [25.0%]	–
Freezing ability	–	2	–	–

^a^Patients could select any reasons that were applicable

^b^Patients with 100% adherence not included

The majority (53%) of participants reported taking capsules three times daily, while only 16% reported taking capsules less than once a day. The majority (67%) of participants reported taking at least four capsules daily, while only two subjects reported taking capsules less than once a day.

The overall incidence of self-reported reasons for missing any of the FOS doses is reported in Table 3, with “Forgot” being the most common reason cited. This was the most common reason for non-adherence in all four groups. In the “With Milk” and “Frozen” groups, the inability to take with milk or take as frozen was the second most common reason for non-adherence in those respective groups.

Self-reported adverse effects that the participant attributed to the FOS were also assessed during the exit interview. The incidence of adverse effect was high across all groups, ranging from 35 to 66%. Only the “No Food” group had > 50% of patients report no adverse effects. There was no difference in the occurrence of any adverse effect among the four groups, including in pairwise comparisons (*p* > 0.1 for all). There was also no difference in frequency of adverse effect occurrence (daily, ≥ 2 times weekly, at least weekly, or less than once weekly). The most common adverse effect attributed to taking FOS was “fishy breath” and was reported in 24 to 43% of patients in any group. Infrequent self-reported adverse effects included upset stomach, heartburn, fishy belching, acne, lower blood pressure, strong taste, and loss of appetite.

## Discussion

In a well-balanced cohort of healthy subjects instructed to take FOS three times daily, the administration method did not significantly impact tolerability or adherence. Adherence was low in general which is to be expected with this type of medication regimen. The “With Milk” group demonstrated a slight trend versus other pairwise comparisons towards improved mean adherence.

The lack of statistically significant results is not surprising considering a number of factors. First, the FOS dose was below the recommended two grams of n3PUFA per day and was also below the prescription dose of 3360 mg daily of omega-3-acid ethyl esters (Lovaza®). Therefore, dose-dependent adverse effects may have occurred less frequently in this study than in studies evaluating higher FOS doses. Secondly, pilot studies tend to enroll a smaller sample size and lack the necessary sample size to meet power. Non-adherence in general occurred which can be expected in a group of healthy subjects that are not accustomed to adhering to a medication schedule, especially a thrice daily regimen.

There are several limitations to the current study. This open-label study enrolled a small sample size in each group and was not powered based on a specific endpoint. A follow-up assessment can be powered appropriately based on the findings in this current study. Efficacy was not assessed. This study was short-term (30 days), and the impact of results on long-term adherence should not be extrapolated. It is not known if manipulation of the FOS itself (e.g., capsule freezing) or absorption method (e.g., concomitant milk ingestion) will affect the stability or effectiveness of FOS in humans. Stability studies should be performed to understand this potential interaction. The significant difference in age between the “With Food” and “No Food” group may have impacted results, but the sample size is too small to compensate for statistically. Pill counts assumed that patients ingested any capsules that were not returned in the bottle at the last visit.

Additional future studies may contemplate several methodology considerations, including FOS purity (amounts of EPA and DHA relative to other oils), FOS dose, and a placebo control. The product used in this study was a relatively low purity product. It has been hypothesized that the higher the product purity, the lower the chance for adverse effects. However, the low purity product used in this study offered the highest potential for adverse effects in the short time period. As previously mentioned, the dose used in this study was lower than those generally recommended for treatment purposes. A higher purity product at higher doses would improve the external validity of any findings. Adding a placebo control is difficult, but it may improve the internal validity of any future outcomes. One of the most commonly reported adverse effects of FOS is the notorious fishy taste and regurgitation. Our findings from the current study did not indicate that freezing the fish oil capsules was significantly able to reduce this unpleasant adverse effect. Future studies may examine the “burpless” fish oil capsules intended to mask this fish taste versus the frozen capsules.

Regarding adverse effect management, the authors did not ascertain whether or not symptoms were self-limiting or improved after discontinuation of the FOS. Adverse effects were self-reported and not assessed by a clinician. These components could be of benefit and should be considered in future studies.

## Conclusion

FOS are a popular source of n3PUFA, but the high pill burden needed to obtain recommended doses is commonly associated with non-adherence and adverse effects. In this randomized pilot clinical trial, the method of administration did not statistically impact rates of adherence or incidence of adverse effects in healthy adults taking fish oil supplement capsules for 30 days. Further investigations are needed to determine if product purity correlates with tolerability. Given the low adherence rates in this study, future studies should be designed to ensure higher adherence rates to improve the probability of any possible differences due to the method of administration.

## Abbreviations

ANOVA: Analysis of variance
DHA: Docosahexaenoic acid
EPA: Eicosapentaenoic acid
FOS: Fish oil supplements
n3PUFA: n-3 long chain polyunsaturated fatty acids
